# Prediction of wheat SPAD using integrated multispectral and support vector machines

**DOI:** 10.3389/fpls.2024.1405068

**Published:** 2024-06-20

**Authors:** Wei Wang, Na Sun, Bin Bai, Hao Wu, Yukun Cheng, Hongwei Geng, JiKun Song, JinPing Zhou, Zhiyuan Pang, SongTing Qian, Wanyin Zeng

**Affiliations:** ^1^ Anyang Institute of Technology, School of Computer Science and Information Engineering, Anyang, China; ^2^ College of Agronomy, High Quality Special Wheat Crop Engineering Technology Research Center, Xinjiang Agricultural University, Urumqi, China; ^3^ Yili Prefecture Institute of Agricultural Science, Yining, China; ^4^ Wheat Research Institute, Gansu Academy of Agricultural Science, Lanzhou, China; ^5^ Cotton Research Institute, Chinese Academy of Agricultural Sciences, Anyang, China

**Keywords:** multispectral, winter wheat, SVM, SPAD, UAV

## Abstract

Rapidly obtaining the chlorophyll content of crop leaves is of great significance for timely diagnosis of crop health and effective field management. Multispectral imagery obtained from unmanned aerial vehicles (UAV) is being used to remotely sense the SPAD (Soil and Plant Analyzer Development) values of wheat crops. However, existing research has not yet fully considered the impact of different growth stages and crop populations on the accuracy of SPAD estimation. In this study, 300 materials from winter wheat natural populations in Xinjiang, collected between 2020 to 2022, were analyzed. UAV multispectral images were obtained in the experimental area, and vegetation indices were extracted to analyze the correlation between the selected vegetation indices and SPAD values. The input variables for the model were screened, and a support vector machine (SVM) model was constructed to estimate SPAD values during the heading, flowering, and filling stages under different water stresses. The aim was to provide a method for the rapid acquisition of winter wheat SPAD values. The results showed that the SPAD values under normal irrigation were higher than those under water restriction. Multiple vegetation indices were significantly correlated with SPAD values. In the prediction model construction of SPAD, the different models had high estimation accuracy under both normal irrigation and water limitation treatments, with correlation coefficients of predicted and measured values under normal irrigation in different environments the value of *r* from 0.59 to 0.81 and *RMSE* from 2.15 to 11.64, compared to *RE* from 0.10% to 1.00%; and under drought stress in different environments, correlation coefficients of predicted and measured values of *r* was 0.69–0.79, *RMSE* was 2.30–12.94, and *RE* was 0.10%–1.30%. This study demonstrated that the optimal combination of feature selection methods and machine learning algorithms can lead to a more accurate estimation of winter wheat SPAD values. In summary, the SVM model based on UAV multispectral images can rapidly and accurately estimate winter wheat SPAD value.

## Introduction

Wheat is one of the most important cereal crops worldwide, and it is heavily dependent on chlorophyll. Chlorophyll is the primary pigment orchestrating photosynthesis. This essential pigment plays a pivotal role in crop growth and nitrogen utilization efficiency by capturing solar energy throughout the process (Lu et al., 2020; Zhang et al., 2022; Yin et al., 2023). Chlorophyll is the basic substance for photosynthesis in green plants and the main photosynthetic pigment in plant leaves. It is an important indicator for studying wheat growth indicators, physiological changes, and nitrogen nutrition status (Jiang et al., 2015; Zhang et al., 2019a). SPAD can directly reflect the chlorophyll content in crop leaves and shows a strong correlation. Numerous studies have shown good consistency between leaf chlorophyll content and SPAD values measured by chlorophyll meters (Sampson et al., 2003; Wang et al., 2022b). Winter wheat is indeed one of the major food crops of the world and plays a significant role in China’s agricultural landscape and the daily lives of people (Liu et al., 2023). Rapid and accurate acquisition of winter wheat SPAD has important scientific significance for agricultural irrigation management, drought monitoring, and crop growth monitoring.

As the winter wheat growth period develops, the canopy reflectance also changes continuously. Spectral indices during the reproductive growth stage of winter wheat have a high correlation with SPAD and can be better used to estimate SPAD, such as spectral vegetation indices during the heading, flowering, and filling stages (Wu et al., 2023). However, as the growth period progresses, the correlation between SPAD and a single-period spectral index reaches a significant level (Ma et al., 2021; Yang et al., 2023), but the process of SPAD formation cannot be determined in a single period. On the contrary, a dataset composed of growth information from multiple growth periods can better reflect crop growth changes and provide more useful information, which is beneficial to improving algorithm estimation accuracy. Crop growth can be predicted by constructing features of nutrient elements and canopy spectra. Therefore, studying the chlorophyll of crops provides a basis for judging the growth situation of crops. Currently, remote sensing technology provides a new solution for estimating crop chlorophyll content (Kaivosoja et al., 2013; Adao et al., 2017; Yang et al., 2019; Liu et al., 2022), and research mainly focuses on predicting chlorophyll content using spectral vegetation indices and spectral information obtained by different sensors combined with ground measurement data.

Remote sensing technology has demonstrated strong competitiveness in precision agriculture under different experimental conditions, especially the convenient application of multispectral imaging technology on UAVs, which has accelerated the development of the technology (Kaivosoja et al., 2013; Yang et al., 2019). The development of UAV technology has greatly facilitated the timely and rapid acquisition of information on crop vegetation, water, soil, and other agricultural and forestry ecological elements, as well as their long-term dynamic monitoring. Compared to data acquisition methods using satellite remote sensing and airborne remote sensing, UAVs have the advantages of maneuverability, flexibility, low data collection costs, and high image resolution. UAV remote sensing imagery is gradually becoming the main data source for the development of smart agriculture and forestry.

By equipping UAVs with hyperspectral cameras, more comprehensive multidimensional image data can be obtained, which enables quantitative inversion of crop phenotypic information such as plant quantity (Font et al., 2014), plant height (Fang et al., 2016), lodging rate (Barata et al., 2024), leaf area index (Liu et al., 2021), chlorophyll content (Kanning et al., 2018), nitrogen element content (Sun et al., 2022; Wang et al., 2022c), pest and disease information (Liu et al., 2020), and other physical and chemical parameters. Compared to RGB three-band image data, hyperspectral imagery provides higher inversion accuracy. However, the high cost, large size, weight, complex data acquisition procedures, and susceptibility to environmental influences restrain the widespread application of hyperspectral imagers. Additionally, acquiring characteristic data on ground-based chlorophyll content often involves destructive methods (Adao et al., 2017). Ground-based data acquisition is limited to a few selected points, making it difficult to represent the characteristics of the entire area. Therefore, the range of traditional ground-based phenotypic data acquisition is inhibited.

Remote sensing data enable high-throughput and large-scale data acquisition, but the influence of spatial image resolution makes it difficult to capture certain local features. Therefore, UAV-based remote sensing technology fills this gap by providing more detailed and localized information for analysis and decision-making in precision agriculture.

Combining ground-based phenotypic data with multispectral imagery from UAVs is an innovative application of UAV multispectral sensors for the estimation of crop chlorophyll content. Machine learning not only enables predictive analysis of traditional data but also demonstrates significant advantages in noise and anomaly handling. Predicting chlorophyll content through the combination of multispectral imagery from UAV remote sensing and machine learning algorithms provides a promising approach. Currently, the integration of machine learning with various vegetation indices has shown strong advantages in agricultural remote sensing. Therefore, this study utilizes high-throughput UAV remote sensing imagery data, along with selected vegetation indices and ground-based SPAD data, to predict the chlorophyll content at different growth stages of winter wheat under various water stresses, aiming to achieve intelligent-level detection of wheat. This paper focuses on the following topics: (1) the impact of chlorophyll content under different irrigation conditions on water response; and (2) the response of the SVM machine learning algorithm in predicting chlorophyll content in winter wheat under different water stress and growth stages. The aim of this study was to explore the potential of SPAD prediction in a large number of multispectral bands and to develop a prediction model to improve the accuracy of SPAD prediction in wheat breeding trials.

## Materials and methods

### Materials

**Figure 1:**
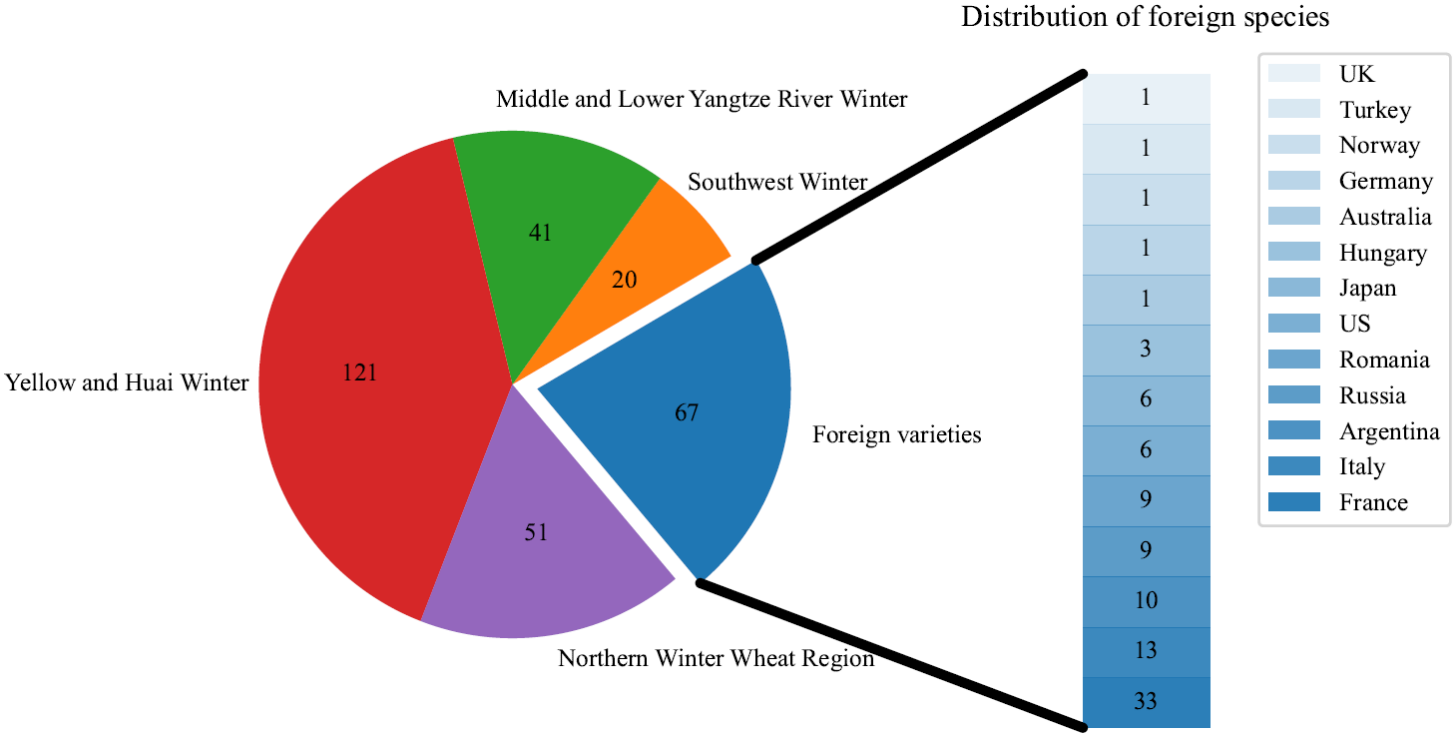
Source distribution of 300 materials.

A total of 300 wheat accessions were used in the experiment, consisting of 65 foreign varieties/lines and 235 varieties/lines from four winter wheat regions in China. These regions included 51 materials from the northern winter wheat region, 121 materials from the Huang Huai winter wheat region, 41 materials from the Yangtze River middle and lower winter wheat regions, and 22 materials from the southwestern winter wheat region. These materials were representative and were provided by the Crop Science Institute of the Chinese Academy of Agricultural Sciences. The distribution of the materials is shown in **Figure 1**.

### Experimental design

The experiment was conducted during the 2020–2022 winter wheat growing season at Manas (86°12’52.2”N, 44°18’15.77”E) and Zepu (77°16’17.22”N, 38°11’21.65”E) in Xinjiang Province, China. The two locations are approximately 1600 kilometers apart, representing the typical geographical differences between northern and southern Xinjiang. Manas has a temperate continental arid to semi-arid climate characterized by cold winters, hot summers, low rainfall, abundant sunshine, high evaporation, and inhibited precipitation. Zepu has a warm temperate continental arid climate, with an average annual temperature of 11.40°C. The extreme maximum temperature reaches 39.50°C, while the extreme minimum temperature can drop to -22.70°C. For this study, two irrigation water stresses were employed: normal irrigation (N) and drought stress (D). Each water treatment included 900 plots, and a total of 300 wheat varieties were selected for the experiment. Each plot was planted with one variety in a completely randomized block design with three replications. The plots were arranged in three rows, with a row length of 2 meters and a row spacing of 20 cm. The drought stress treatment involved water control management during the wheat heading, flowering, filling, and maturity stages. Field management practices followed the local conventional cultivation methods, including fertilization, drip irrigation, pest control, and weed management. The wheat planted grew normally, and the field conditions were favorable.

### Image acquisition

The UAV used in this study was the DJI Phantom 4 Multispectral (P4M) drone. This device was equipped with one visible light sensor channel and five multispectral sensor channels, which capture data in five different wavelength bands: 475 ± 16nm (blue), 560 ± 16nm (green), 668 ± 16nm (red), 717 ± 16nm (red edge), and 840 ± 26nm (NIR). The high-definition digital camera model was the FC 6310. Its main parameters were: a 1-inch CMOS sensor with effective pixels of 20 million, a resolution of 5472 × 3648, an aperture value of f/5.6, and a focal length of 9 mm. Each flight captured six images, each with a pixel resolution of over two million. The P4M drone was equipped with the TimeSync system, which ensured centimeter-level positioning accuracy. Additionally, it has a light intensity sensor integrated on the top to capture solar irradiance data for radiometric calibration. This helped in compensating for the effects of environmental light and improved the accuracy and consistency of the collected data at different times. During the acquisition of multispectral images, the UAV was flown autonomously along predefined flight paths under clear and windless conditions at noon. The multispectral camera lens was oriented vertically downward. The flight parameters were presented in **Table 1**.

**Table 1:** Parameters of UAV multispectral image acquisition.

Parameter	Parameter values
Flight altitude	12m
Flight Speed	5.4km/h
Course overlap ratio	75%
Lateral overlap rate	75%
Spectral type	Blue、Green、Red、Red_edge、Nir

### Data acquisition plan

The data collection involved the measurement of SPAD values in the canopy of winter wheat and the acquisition of multispectral imagery using the UAV. The SPAD measurements and multispectral imagery data were collected at three different growth stages of winter wheat: heading, flowering, and filling. Additionally, the data collection plan included two different watering treatments. The specific data collection plan for SPAD measurements and UAV multispectral imagery was provided in **Table 2**.

**Table 2:** UAV Multispectral Imagery and SPAD data acquisition program.

Collection time	Environment	Fertility period	Collection data
2021.5.08	Manas	heading	Photosynthetic traits multispectral imaging
2021.5.21		flowering	
2021.5.28		filling	
2021.4.30	Zepu	heading	
2021.5.08		flowering	
2021.5.15		filling	
2022.5.13	Manas	heading	
2022.5.20		flowering	
2022.5.27		filling	
2022.4.27	Zepu	heading	
2022.5.08		flowering	
2022.5.16		filling	

### Determination of chlorophyll content

The measurements were taken during three different growth stages of wheat: heading, flowering, and filling. On the day of the UAV flight, the relative chlorophyll content of different genotypes was synchronously measured. Five wheat plants with consistent growth were selected from each variety. The SPAD-502 Plus chlorophyll meter, manufactured by the Japanese company Minolta, was used for measurements. This instrument has been widely used by many researchers to obtain SPAD data for wheat (Zhang et al., 2019b; Wang et al., 2022a). In the experimental field, the upper, middle, and lower leaves of winter wheat plants were selected, and SPAD values were recorded. The average chlorophyll content of the three leaf positions was considered the canopy SPAD value of the respective winter wheat plant. Then, the average SPAD value of five winter wheat plants was calculated as the canopy SPAD value for that specific variety of winter wheat.

### Image processing

**Figure 2:**
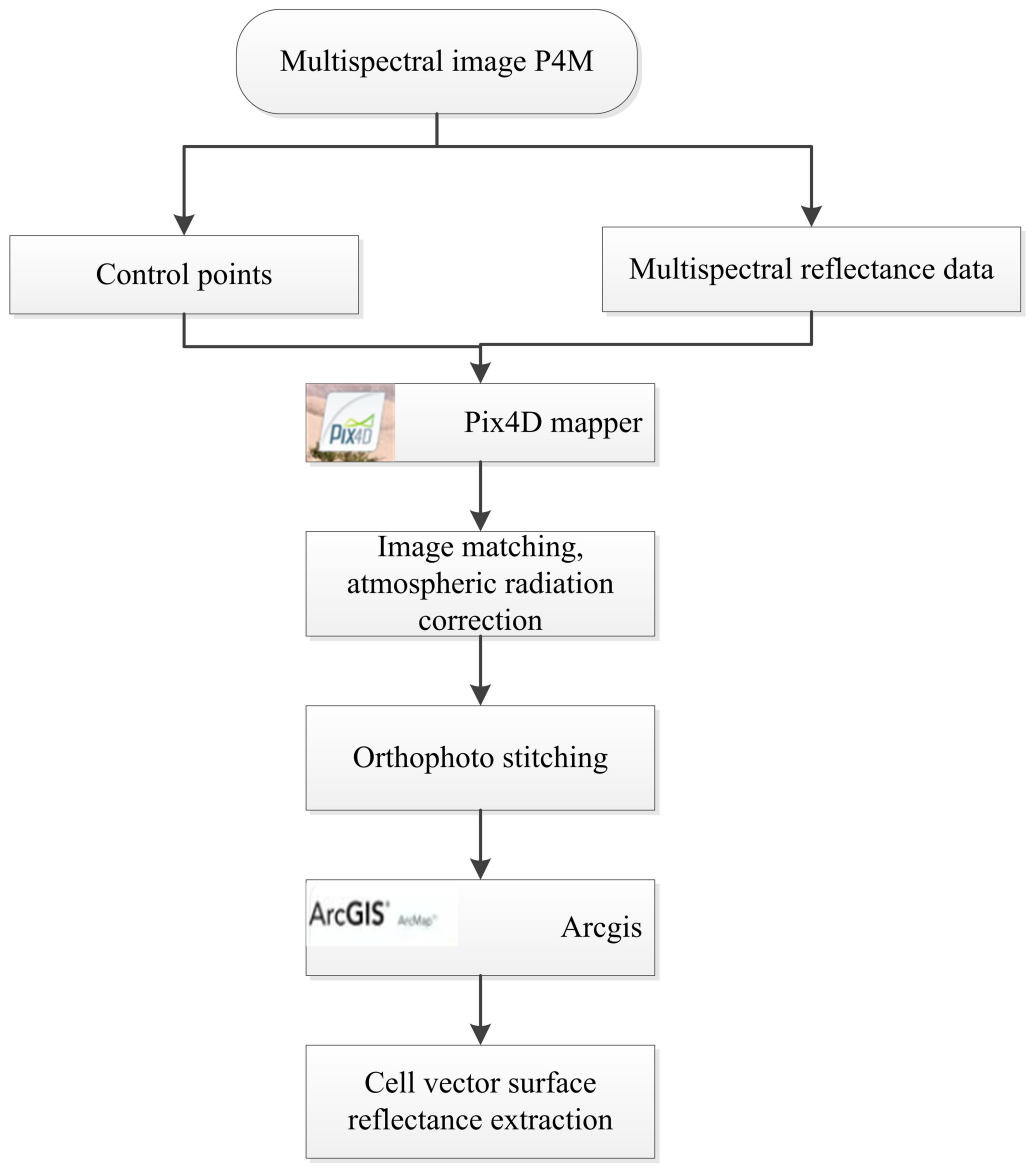
Flow chart of UAV image processing.

In this study, the DJI P4M drone was used to capture images. The acquired raw images were processed using Pix4Dmapper software (https://pix4d.com/) for image stitching. Prior to the stitching process, image correction was performed based on ground control points to generate a Digital Orthophoto Map (DOM). Subsequently, reflectance conversion was conducted using white balance correction to convert pixel values to reflectance, resulting in reflectance images for all spectral channels. Finally, the ARCGIS software (Version 10.3.1, Esri, USA) (http://www.esri.com/arcgis/about-arcgis) was employed to extract vector surfaces of the study area, enabling the acquisition of reflectance data for further vegetation index calculations. The processing flow of the image is shown in **Figure 2**.

### Selection of vegetation indices

Combining reflectance values from different spectral bands forms vegetation indices, which can reduce the influence of factors such as background soil on vegetation spectra to some extent, thereby improving the accuracy of estimating chlorophyll content. In this study, various vegetation indices were selected and then evaluated in combination with the correlation between vegetation indices and SPAD values. The chosen vegetation indices were used to inversely model the SPAD values and make predictions. The formulas for calculating vegetation indices are presented in **Table 3**.

**Table 3:** Vegetation index and its calculation formula.

Vegetation index	formula to calculate	Reference
NDVI	NDVI=(RNir−RRed)/(RNir+RRed)	(Rouse et al., 1973)
GNDVI	$GNDVI=(R_{Nir}-R_{Green})/(R_{Nir}+R_{Green})$	(Wagner, 1996)
NGBDI	$NGBDI=(R_{Green}-R_{Blue})/(R_{Green}+R_{Blue})$	(Dong et al., 2015)
NGRDI	NGRDI=(RGreen−RRed)/(RGreen+RRed)	(Dong et al., 2015)
RERDVI	$RERDVI=(R_{Nir}-R_{Red\_edge})/(R_{Nir}+R_{Red\_edge})$	(Xue and Su, 2017)
SAVI	$SAVI=2.5*(R_{Nir}-R_{Red})/(R_{Nir}+R_{Red}+0.5)$	(Huete, 1988)
OSAVI	$OSAVI=(R_{Nir}-R_{Red})/(R_{Nir}+R_{Red}+0.16)$	(Rondeaux et al., 1996)
RVI	$RVI=R_{Nir}/R_{Red}$	(Wang et al., 2007)
DVI	$DVI=R_{Nir}-R_{Red}$	(Tucker, 1979)
GRVI	$GRVI=R_{Nir}/R_{Green}$	(Tucker, 1979)

$R_{Blue}$ 、 $R_{Green}$ 、 $R_{Red}$ 、 $R_{Red\_edge}$ and $R_{Nir}$ respectively represent the reflectance of the blue wave band, green band, red band, red edge band, and near infrared band.

### Analytical methods

For this study, the classic SVM model, a machine learning algorithm, was selected. The SVM model consists of two main components: data construction and model creation. In the data construction phase, X represents the input variables (vegetation index combinations) and Y represents the target variable (measured values of the studied photosynthetic trait). Assuming Y is a continuous variable and the target value, the training dataset D is as shown in Equation (1):


(1) $$D=\{(x_{1},y_{1}),(x_{2},y_{2}),\ldots ,(x_{N},y_{N})\}$$

(2) $$x_{i}=(x_{i}^{\left(1\right)},x_{i}^{\left(2\right)},\ldots ,x_{i}^{\left(n\right)})(i=1,2,\ldots ,N)$$

*x_i_* represents the feature vector (Equation 2), *n* represents the number of features, and *N* represents the material capacity, which in this research corresponds to the number of residential areas. A heuristic method is adopted for partitioning the feature space. Each time, the current set of features and their values are examined one by one. The optimal split point is selected based on the criterion of minimizing the squared error. For example, for the j feature variable $x^{\left(j\right)}$ in the training set and its set of values *S*, it is used as the splitting variable to define two regions $R_{1}(j,s)=\{x|x^{\left(j\right)}\leq \text{s}\}$ and $R_{2}(j,s)=\{x|x^{\left(j\right)}\text{>s}\}$ and find the optimal *j* and *s*.

In this study, the SVM kernel used for regression analysis is support vector machine regression (SVR), which establishes the dependency between vegetation indices and photosynthetic traits. The set *X* mentioned above represents the vegetation indices, while *Y* represents the photosynthetic traits. To achieve higher accuracy and faster convergence, the data is first subjected to standardization before the model regression analysis. Let us assume *fin_ij_* represents the *j* feature value of the *i* material, *fin_min,j_* represents the minimum value of the *j* feature, and *fin_max,j_* represents the maximum value of the *j* feature. The standardized result *fin_xj_* is calculated as shown in Equation (3):


(3) $$fin_{ij}=\frac{fin_{\text{ij}}-fin_{\min,\text{j}}}{fin_{\max,j}-fin_{\min,j}}$$

This equation calculates the standardized value of each feature by subtracting the minimum value and dividing it by the range (maximum value minus minimum value) of that feature. Standardization was performed to ensure that all features were on the same scale, which helped in comparison and analysis. It enabled a fair assessment of the relative importance of different features and prevented any bias that may have arisen from the original scale of the data.

### Indicators for model evaluation

During the modeling process, it was important to ensure that the training set and validation set did not overlap. Finally, the coefficient of determination (*r*), root mean square error (*RMSE*), and relative error (*RE*) are used as indicators to assess the correlation of the model’s predicted values.


(4) $$\text{r}=\frac{\sum_{i}^{n}=1{\left({\hat{y}}_{i}-\bar{y}\right)}^{2}}{\sum_{i}^{n}=1{\left(y_{i}-\bar{y}\right)}^{2}}$$

(5) $$\text{RMSE}=\sqrt{\frac{\sum_{i}^{n}=1{\left({\hat{y}}_{i}-y_{i}\right)}^{2}}{n}}$$

(6) $$\text{RE=}\left|\frac{y_{i}-{\hat{y}}_{i}}{y_{i}}\right|$$

In Equations (4–6) of the given expression, ${\hat{y}}_{i}$ represents the predicted values of the photosynthetic trait; $y_{i}$ represents the measured values of the photosynthetic trait; $\bar{y}$ represents the average value of the photosynthetic trait; and *n* represents the number of validation materials. These equations are utilized to calculate and evaluate various metrics that assess the performance of the model’s predictions in comparison to the actual measurements of the photosynthetic trait.

### Modeling framework

**Figure 3:**
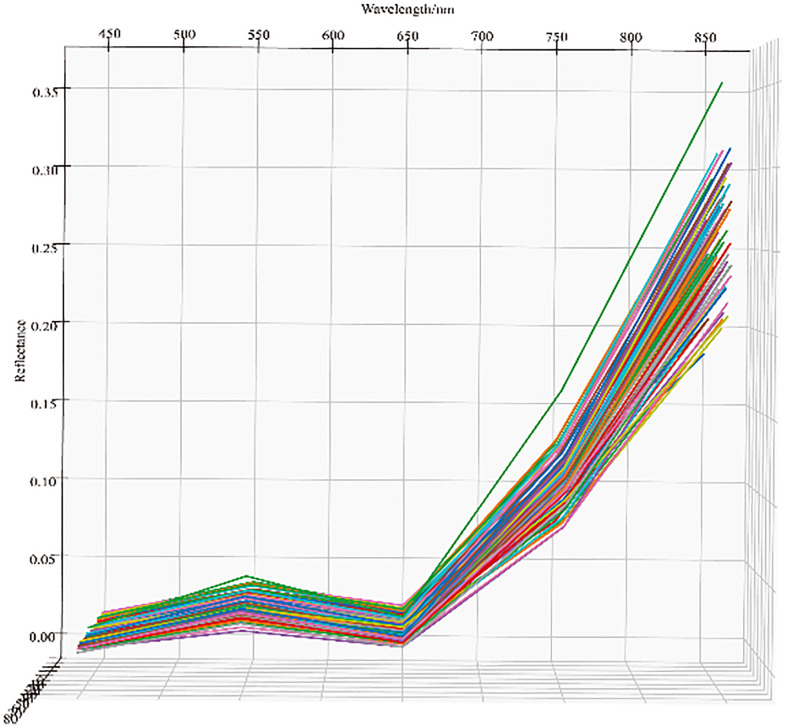
The data processing procedure and the ensemble SVM model averaging framework.

The model construction flow of this study is shown in **Figure 3**. SPAD used 1,800 materials of water and drought treatments at the tasseling, flowering, and grouting stages in 2021 and 900 materials of water and drought treatments at the tasseling, flowering, and grouting stages in 2022 from the Manas and Zepu regions in Xinjiang for the construction and prediction of the model. SPAD prediction model for different periods of data with different treatments, data in accordance with the ratio of 7:3 for the division of training set and test set, test set 210 materials, test set 90 materials, validation, and finally in the BLUP calculation of all the data and use all the data as a training set, through the original data calculated BLUP values and the model prediction of the BLUP to model the Evaluation.

## Results

### Accuracy evaluation of UAV multispectral image data

**Figure 4:**
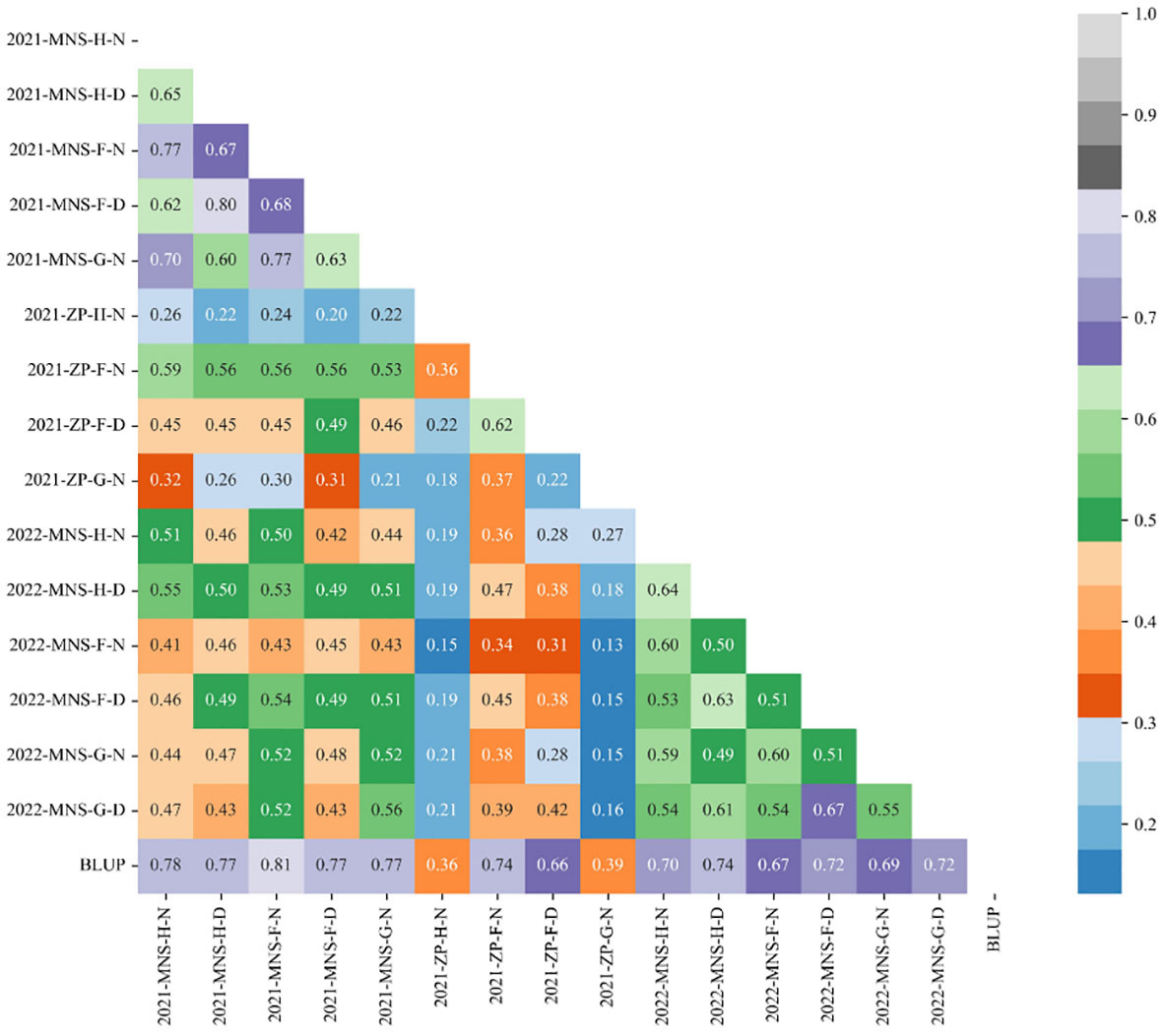
Reflectance curves of the five bands of the multispectrum under different treatments in different fertility periods in different environments.

The winter wheat UAV remote sensing images obtained during the heading, flowering, and filling stages under normal irrigation and drought stress treatments in Manas and Zepu regions in Xinjiang from 2020 to 2022 were processed. Reflectance images of different bands from different zones were obtained. The distribution trend of reflectance is shown in **Figure 4**. From the figure, it can be observed that there was a distinct peak approximately 550 nm and a pronounced trough at 650 nm. After 750 nm, the reflectance curve becomes steeper, indicating a rapid increase in reflectance. The obtained reflectance values in this study are consistent with previous research (Fu et al., 2019). Moreover, the results demonstrate that the reflectance of the multispectral data has high accuracy within the 466 nm–830 nm spectral range. This finding is consistent with Aasen’s study (Aasen et al., 2015). Additionally, the five selected multispectral bands in this study fall within this spectral range, which enables the estimation of canopy chlorophyll content in winter wheat.

### Distribution of winter wheat canopy SPAD phenotypes

**Figure 5:**
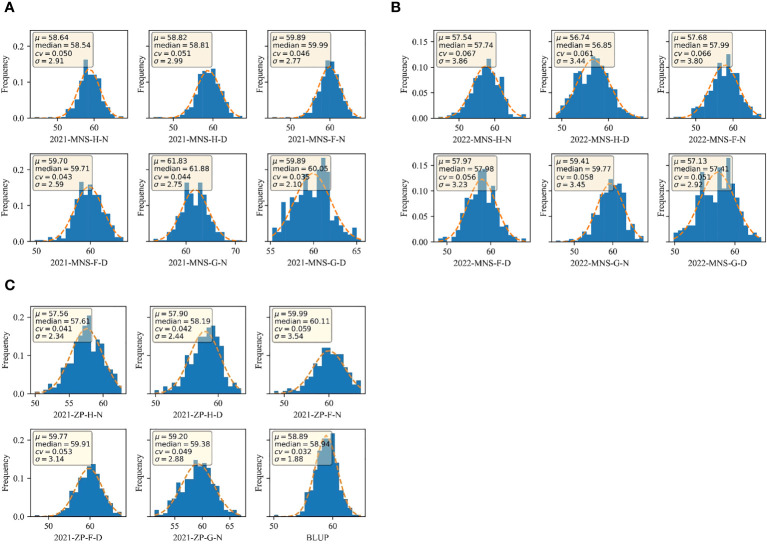
SPAD distribution of BLUP under different treatments at different fertility stages in different environments and the full average environment **(A)** SPAD distribution under normal irrigation and drought stress at full fertility in 2021 MNS; **(B)** SPAD distribution under normal irrigation and drought stress at full fertility in 2022 MNS; **(C)** SPAD under normal irrigation and drought stress at full fertility in 2021 ZP and BLUP distributions for all environments. 2021-MNS-H-N: 2021 Manas normal irrigation at heading stage; 2021-MNS-H-D: 2021 Manas drought stress at heading stage; 2021-MNS-F-N: 2021 Manas normal irrigation at the flowering stage; 2021-MNS-F-D: 2021 Manas drought stress at the flowering stage; 2021-MNS-G-N: 2021 Manas normal irrigation at the stage of filling; 2021-MNS-G-D: 2021 Manas drought stress at the stage of filling; 2022-MNS-H-N: 2022 Manas normal irrigation at the stage of heading; 2022-MNS-H-D: 2022 Manas drought stress at the stage of heading; 2022-MNS-F-N: 2022 Manas normal irrigation at the stage of flowering irrigation; 2022-MNS-F-D: 2022 Manas flowering drought stress; 2022-MNS-G-N: 2022 Manas filling drought stress; 2022-MNS-G-D: 2022 Manas filling drought stress; 2021-ZP-H-N: 2021 ZP heading normal irrigation; 2021-ZP-H-D: 2021 ZP heading stage drought stress; 2021-ZP-F-N: 2021 ZP flowering stage normal irrigation; 2021-ZP-F-D: 2021 ZP flowering stage drought stress; 2021-ZP-G-N: 2021 ZP grubbing stage normal irrigation; BLUP: best linear unbiased prediction for the whole environment.

In this study, we investigated the SPAD traits of Manas (MNS) wheat in Xinjiang, China, during the reproductive stages of heading, flowering, and filling under both normal irrigation and drought stress conditions in the years 2020–2022. We also examined the SPAD traits of Zepu (ZP) wheat in Xinjiang during the heading, flowering, and filling stages under normal irrigation conditions in 2021. We evaluated the SPAD traits from four dimensions, namely the mean represented by *μ*, median represented by median, coefficient of variation represented by *CV*, and standard deviation represented by *σ*. From **Figure 5A**, it can be observed that during the heading stage of Manas wheat in 2021, the *CV* of SPAD traits was 5.00% under normal irrigation and 5.10% under drought stress. Similarly, during the flowering stage, the *CV* was 4.60% under normal irrigation and 4.30% under drought stress. For the grain filling stage, the *CV* was 4.40% under normal irrigation and 3.50% under drought stress. Considering the entire reproductive period, the *CV* ranged from 4.40% to 5.00% under normal irrigation and from 3.50% to 5.10% under drought stress. Additionally, the mean (*μ*) values during the entire reproductive period ranged from 58.64 to 61.83, while under drought stress, the range was 58.82–59.89. Similarly, from **Figure 5B**, it can be seen that during the heading stage of Manas wheat in 2022, the *CV* of SPAD traits was 6.70% under normal irrigation and 6.10% under drought stress. During the flowering stage, the *CV* was 6.60% under normal irrigation and 5.60% under drought stress. For the filling stage, the *CV* was 5.80% under normal irrigation and 5.10% under drought stress. Considering the entire reproductive period, the *CV* ranged from 5.80% to 6.70% under normal irrigation and from 5.10% to 6.10% under drought stress. Additionally, the mean (*μ*) values during the entire reproductive period ranged from 57.54 to 59.41, while under drought stress, the range was 56.74–57.98.

From **Figure 5C**, it can be observed that during the heading stage of Zepu wheat in 2021, the coefficient of variation (*CV*) of SPAD traits was 4.10% under normal irrigation and 4.20% under drought stress. During the flowering stage, the *CV* was 5.90% under normal irrigation and 5.30% under drought stress. For the grain filling stage, the *CV* was 4.90% under normal irrigation. Considering the entire reproductive period, the *CV* ranged from 4.10% to 5.90% under normal irrigation and from 4.20% to 5.30% under drought stress. Additionally, the mean (*μ*) values during the entire reproductive period ranged from 57.56 to 59.99, while under drought stress, the range was 57.90–59.77.

Overall, the data shows a large dispersion and variation range, indicating significant variation in SPAD traits during the heading, flowering, and filling stages of Zepu wheat. This also suggests that the population exhibits abundant genetic variation, consistent with typical quantitative trait inheritance characteristics, and the data follows a continuous and normal distribution. From the perspective of normal irrigation and drought stress, the range of average SPAD values throughout the reproductive period indicates that SPAD content is higher under normal irrigation compared to drought stress. Furthermore, considering the *CV*, there is minimal difference in heading stage *CV* between normal irrigation and drought stress across different environments and reproductive stages. However, with the progression of the reproductive stage and prolonged drought stress, the variability range under normal irrigation was higher than that under drought stress. As the reproductive stage continues, the overall variability range gradually narrows.

### SPAD correlation analysis of winter wheat canopy in different environments

**Figure 6:**
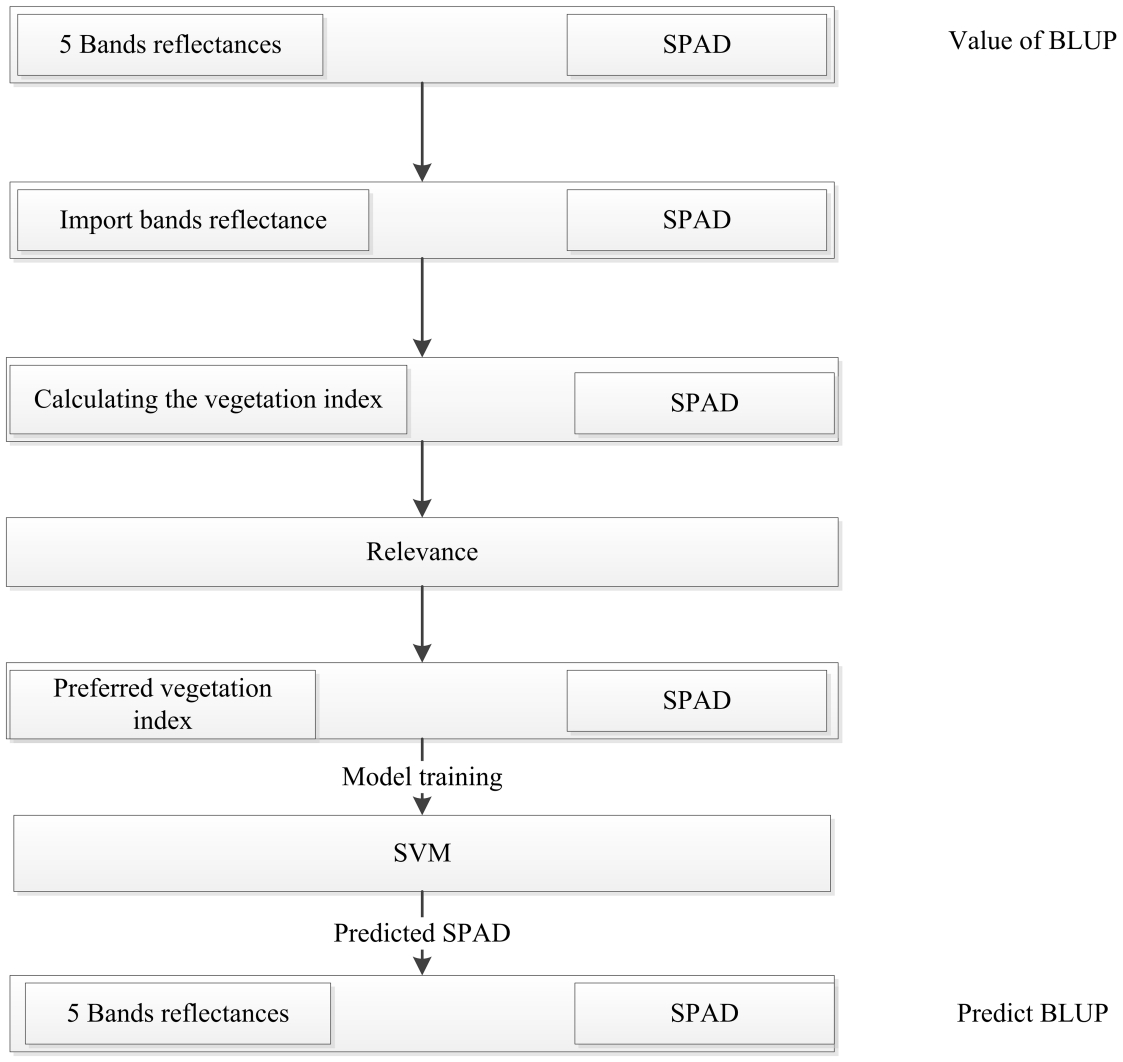
Correlation of SPAD under different treatments at different fertility stages in different environments and the value of BLUP. Same as all the notes in **Figure 4**.

The correlation analysis results of crown SPAD content at the heading, flowering, and filling stages of winter wheat under normal irrigation and drought stress treatments in Manas and Zepu regions of Xinjiang from 2020 to 2022 are shown in **Figure 6**.

In the figure, the correlation of crown SPAD content at different growth stages of winter wheat was compared, and it can be observed from **Figure 6** that the crown SPAD content of winter wheat had reached a significant level. In the Manas environment of 2021, the correlation under normal treatments at different growth stages ranged from 0.21 to 0.81, while under drought stress treatments, the correlation ranged from 0.20 to 0.77. In the Zepu environment of 2021, the correlation under normal treatments at different growth stages ranged from 0.13 to 0.74, while under drought stress treatments, the correlation ranged from 0.22 to 0.66. In the Manas environment of 2022, the correlation under normal treatments at different growth stages ranged from 0.51 to 0.70, while under drought stress treatments, the correlation ranged from 0.49 to 0.74. Overall, the correlation of crown SPAD content in winter wheat under different environmental conditions and treatments ranged from 0.13 to 0.81, reaching a significant level.

### Evaluation of the accuracy of the prediction model

The SPAD values of winter wheat during three growth stages (heading, flowering, and filling) under two water stresses were modeled using the SVM machine learning algorithm, and the results are shown in **Table 4**. In the Zepu region of Xinjiang, under normal irrigation during the heading stage, the predicted values showed a correlation coefficient (*r*) of 0.73 with the measured values. The root mean square error (*RMSE*) was 6.07, and the relative error (*RE*) was 0.10%. For the flowering stage with normal irrigation, the corresponding values were *r* = 0.73, *RMSE* = 5.87, and *RE* = 0.20%. During the filling stage with normal irrigation, the values were *r* = 0.73, *RMSE* = 6.01, and *RE* = 0.20%. In the Manas region, during the heading stage with normal irrigation, the predicted values showed a correlation coefficient ranging from 0.59 to 0.80. The *RMSE* ranged from 2.51 to 7.95, and the *RE* ranged from 0.20% to 0.80%. For the flowering stage with normal irrigation, the corresponding values were *r* = 0.65–0.81, *RMSE* = 2.15–11.64, and *RE* = 0.10%–1.20%. During the grain filling stage with normal irrigation, the values were *r* = 0.59–0.81, *RMSE* = 4.87–10.05, and *RE* = 0.10%–0.40%. Considering both the Zepu and Manas regions over 2 years, during the heading stage with normal irrigation, the predicted values showed a correlation coefficient ranging from 0.69 to 0.80. The *RMSE* ranged from 2.51 to 7.95, and the *RE* ranged from 0.10% to 0.80%. For the flowering stage with normal irrigation, the corresponding values were *r* = 0.65–0.81, *RMSE* = 2.15–11.64, and *RE* = 0.10%–1.20%. During the grain filling stage with normal irrigation, the values were *r* = 0.59–0.81, *RMSE* = 4.87–10.05, and *RE* = 0.20%–1.00%. In both the Zepu and Manas regions of Xinjiang over the course of 2 years, during the heading stage under drought stress, the predicted values showed *r* from 0.69 to 0.77. The *RMSE* ranged from 2.48 to 12.6, and the *RE* ranged from 0.20% to 0.80%. For the flowering stage under drought stress, the corresponding values were *r* = 0.71–0.79, *RMSE* = 2.30–7.72, and *RE* = 0.10%–0.80%. During the grain filling stage under drought stress, the values were *r* = 0.70–0.73, *RMSE* = 7.90–9.50, and *RE* = 0.60%–1.30%.

**Table 4:** Model analysis of three fertility SVM models combined with vegetation indices to predict SPAD.

Fertility	Environments	Treatment	*r*	*RMSE*	*RE(*%)
heading	2021-ZP	N	0.73	6.07	0.10
		D	0.76	12.60	0.80
	2021-MNS	N	0.80	2.51	0.20
		D	0.77	2.48	0.20
flowering	2021-ZP	N	0.73	5.87	0.20
		D	0.79	4.70	0.30
	2021-MNS	N	0.81	2.15	0.10
		D	0.75	2.30	0.10
filling	2021-ZP	N	0.73	6.01	0.20
		D	0.75	12.94	0.70
	2021-MNS	N	0.59	4.87	0.40
		D	0.70	9.50	1.30
heading	2022-MNS	N	0.69	7.95	0.80
		D	0.69	7.97	0.50
flowering	2022-MNS	N	0.65	11.64	1.20
		D	0.71	7.72	0.80
filling	2022-MNS	N	0.81	10.05	1.00
		D	0.73	7.90	0.60

2021-ZP: 2021 Zepu environment; 2021-MNS: 2021 Manas environment; 2022-ZP: 2022 Zepu environment; 2022-MNS: 2022 Manas environment; N: normal irrigation treatment; D: drought stress treatment.

**Figure 7:**
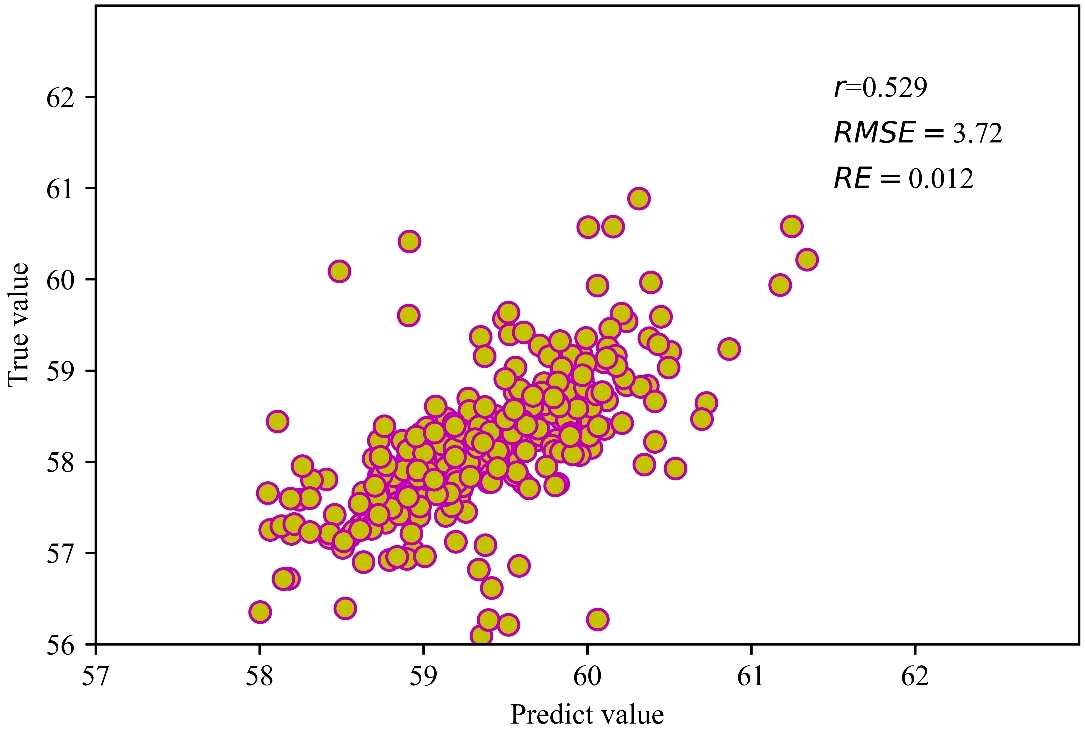
Distribution of BLUP values for predicted and tested values in all environments.

The BLUP values obtained through the best linear unbiased prediction calculation for the SPAD measurements and predicted values in all environments are shown in **Figure 7**. The BLUP model achieved *r*= 0.53, *RMSE*=3.72, and *RE*= 1.20%. In terms of prediction accuracy, the model performed better for normal irrigation conditions compared to drought-stress conditions. Overall, the majority of the predictions had an accuracy of 0.60 or higher, indicating that the model’s predictive performance was quite satisfactory.

## Discussion

### Effect of drought treatment on chlorophyll

The impact of drought on crops was widespread, particularly for high-yielding and stable-yielding crops, with recent years in China witnessing a reduction in crop production by 100 million metric tons due to drought (Wang et al., 2003). Moreover, extreme weather events have become increasingly frequent, significantly affecting the entire growth stage of wheat under drought stress. Additionally, the experimental area in this study primarily focused on the Xinjiang region, which experiences perennial drought stress on crops. Therefore, studying drought stress in the Xinjiang region holds greater significance for crop productivity. Referring to the historical research conducted on wheat and its growth stages, the entire growth period can be categorized into four stages: the initial stage (sowing-emergence), the development stage (emergence-heading), the middle stage (heading-maturity), and the late stage (maturity-harvest). Previous studies on the effects of drought stress at different growth stages of wheat suggest that it can exert varying degrees of influence on the physiological and biochemical phenotypes within the plant. These changes manifest in the alterations of SPAD content and leaf area index (LAI), indicating a close relationship between chlorophyll content and the drought resistance and yield traits of wheat (Li et al., 2010).

Through the study of the photosynthetic characteristics of wheat under normal irrigation and drought stress across multiple environments and growth stages, the analysis of SPAD values indicates that the average photosynthetic content under normal irrigation is higher than that under drought stress throughout the entire growth period. Furthermore, as the growth stage progresses, the differences in SPAD values between the two conditions become smaller. From a physiological perspective, once wheat enters the heading stage, it requires ample water for vigorous growth and to effectively capture a large area of sunlight for photosynthesis and dry matter accumulation. As the growth stage extends, the crop gradually enters the senescence phase, resulting in a weakened photosynthetic capacity and reduced accumulation of dry matter, leading to decreased variation in the measured values (Wang, 2008). In addition, drought stress also affects the physiological and biochemical processes within plants. Previous studies have shown that drought stress leads to stomatal closure and a decrease in stomatal conductance, resulting in reduced photosynthesis and dry matter production. Drought stress also leads to the accumulation of malondialdehyde (MDA) and peroxidase (POD) in plants. MDA is an active lipid peroxidation product that can crosslink and aggregate lipids, nucleic acids, and proteins and affect the composition of cell membrane components, including chloroplast membrane formation. POD, under prolonged drought stress, generates reactive oxygen species and triggers lipid membrane peroxidation. Both substances can cause changes in membrane structure, resulting in leaf dehydration and affecting water metabolism, thereby influencing crop yield. These findings are consistent with the decreasing trend of wheat’s photosynthetic characteristics under drought stress, as observed in this study (Wang et al., 2003).

### Generalization of SPAD inversion models

In this study, a support vector machine (SVM) regression model was developed using multiple vegetation indices to estimate SPAD values. Experimental results demonstrated that the SVM model exhibited certain advantages during the heading, flowering, and filling stages. The generalizability of the regression model was validated using the SVM algorithm on a dataset of 300 natural populations. Before conducting the model inversion, reflectance values were extracted from different spectral bands of the acquired multispectral data. The analysis of the reflectance extraction results showed distinct peaks and valleys in the data, which were consistent with the findings of Aasen et al (Aasen et al., 2015). This consistency indicates the accuracy of the obtained reflectance data and prepares the groundwork for subsequent model inversion processes.

In the same way, it was challenging to infer the distribution of SPAD values using a single vegetation index. Additionally, incorporating too many vegetation indices in the calculation process exponentially increases the complexity. To address this, the present study employed a comprehensive screening and evaluation approach by considering both the contribution rate of vegetation indices and their correlation with SPAD values. As a result, different vegetation indices were selected for model construction during various growth stages. Based on the experimental results, it can be observed that the SVM model performed well under normal irrigation conditions during the heading, flowering, and grain-filling stages of wheat in the Zepu region. The *r* between the predicted and measured values was 0.73, with an *RMSE* of 6.07 and a *RE* of 0.10% during the heading stage. During the flowering stage, the *r* was 0.73, the *RMSE* was 5.87, and the *RE* was 0.20%. For the filling stage, the *r* was 0.73, the *RMSE* was 6.01, and the *RE* was 0.20%. In the Manas region, under normal irrigation during the heading stage, the range of *r* values between the predicted and measured values was 0.59–0.80, the *RMSE* ranged from 2.51 to 7.95, and the *RE* ranged from 0.20%–0.80%. During the flowering stage, the *r* ranged from 0.65–0.81, the *RMSE* ranged from 2.15 to 11.64, and the *RE* ranged from 0.10%-1.20%. For the filling stage, the range of *r* values was 0.59–0.81, the *RMSE* ranged from 4.87–10.05, and the *RE* ranged from 0.10%–0.40%. In both the Zepu and Manas regions, during the heading stage of wheat cultivation across 2 years under normal irrigation, the range of *r* values between the predicted and measured values was 0.69–0.80, the *RMSE* ranged from 2.51 to 7.95, and the *RE* ranged from 0.10%–0.80%.

## Conclusion

In this study, a method utilizing UAV remote sensing imagery is developed for estimating the SPAD of wheat canopies. The SVM machine learning algorithm was employed for model inversion. Under normal irrigation during the booting stage, the *r* between predicted and measured values ranged from 0.69 to 0.80, with *RMSE* ranging from 2.51 to 7.95 and *RE* ranging from 0.10% to 0.80%. During the flowering stage under normal irrigation, the *r* values ranged from 0.65 to 0.81, the *RMSE* ranged from 2.15 to 11.64, and the *RE* ranged from 0.10% to 1.20%. During the grain filling stage under normal irrigation, the *r* values ranged from 0.59 to 0.81, the *RMSE* ranged from 4.87 to 10.05, and the *RE* ranged from 0.20% to 1.00%. Under water stress during the booting stage, the *r* values between predicted and measured values ranged from 0.69 to 0.77, with *RMSE* ranging from 2.48 to 12.60 and *RE* ranging from 0.20% to 0.80%. Under water stress during the flowering stage, the *r* values ranged from 0.71 to 0.79, the *RMSE* ranged from 2.30 to 7.72, and the *RE* ranged from 0.10% to 0.80%. Under water stress during the grain filling stage, the *r* values ranged from 0.70 to 0.75, the *RMSE* ranged from 7.90 to 12.94, and the *RE* ranged from 0.50% to 1.30%. The BLUP (Best Linear Unbiased Prediction) values for all SPAD tests and predicted values under all conditions resulted in an *r* value of 0.53, an *RMSE* of 3.72, and a *RE* of 1.20%. In terms of prediction accuracy, the models performed better under normal irrigation compared to water stress conditions, with most of the overall prediction accuracy being above 0.60. This study’s results demonstrate that the SVM models constructed for different growth stages and water stress can effectively estimate the chlorophyll content in winter wheat canopies with varying levels of growth vigor.

## Data availability statement

The original contributions presented in the study are included in the article/supplementary material. Further inquiries can be directed to the corresponding author.

## Author contributions

WW: Formal analysis, Writing – original draft, Writing – review & editing. NS: Formal analysis, Writing – original draft, Writing – review & editing. BB: Conceptualization, Project administration, Resources, Supervision, Writing – original draft, Writing – review & editing. HW: Data curation, Investigation, Methodology, Writing – review & editing. YC: Data curation, Investigation, Methodology, Writing – review & editing. HG: Conceptualization, Project administration, Resources, Supervision, Writing – review & editing. JS: Data curation, Investigation, Methodology, Writing – review & editing. JZ: Data curation, Investigation, Methodology, Writing – review & editing. ZP: Data curation, Investigation, Methodology, Writing – review & editing. SQ: Writing – review & editing. WZ: Data curation, Investigation, Methodology, Writing – review & editing.
